# Chinese preschoolers’ ownership reasoning based on first possession heuristic

**DOI:** 10.1371/journal.pone.0260335

**Published:** 2021-12-02

**Authors:** Zhanxing Li, Xiaoli Ni, Liqi Zhu, Jing Li

**Affiliations:** 1 Institute of Social Psychology, School of Humanities and Social Sciences, Xi’an Jiaotong University, Xi’an, China; 2 CAS Key Laboratory of Behavioral Sciences, Institute of Psychology, Chinese Academy of Sciences, Beijing, China; 3 Department of Psychology, University of Chinese Academy of Sciences, Beijing, China; 4 Law School, Kunming University of Science and Technology, Kunming, China; University of Maryland, UNITED STATES

## Abstract

First possession is a common heuristic people use to solve property conflicts. Previous studies examined whether young children judged ownership based on the first possession heuristic and its stability when conflicting with other cues such as labor, but few focused on the effects in the discovery context. In this study, we used two discovery stories which indicate the discovered object was not owned by anyone beforehand and investigated ownership reasoning with the first possession heuristic in Chinese 3- to 6-year-old preschoolers. By pitting the first possession cue against the labor cue, we investigated the stability of the first possession heuristic in young children’s ownership reasoning. The results showed that in the condition where there was only the first possession cue, both the younger and older groups used the first possession heuristic to reason about ownership. However, in the labor condition, 5- and 6-year-olds ceased to support the first possessor and turned to assign objects to the laborer, whereas 3-year-old children still insisted on the first possession heuristic (Study 1 and Study 2). Children across four age groups did not assign ownership to the person who just played with the object but did not modify it (Study 2). The results demonstrate that Chinese preschoolers understand the role of first possession in ownership assignment at an early age in the discovery context but the elderly preschoolers do not rely on the first possession cue when there are conflicting cues such as labor.

Ownership represents a specific relationship between the agent and property, which stipulates that only the owners have rights to use, change, transfer and track the property, and other agents do not have these rights [1–5]. It was found that disputes over objects are some of the earliest and most frequent and intense conflicts among children [6–9]. Even for adults, complex ownership problems sometimes happen and cannot be resolved by one arbitrary rule [10, 11]. To reduce social conflict and make their resolutions more acceptable, human society has agreed on some basic principles concerning ownership. Among these principles, first possession is very common and often used in young children’s life [12–14].

Imagine two people who find a beautiful shell alongside the sea. According to the first possession rule, the shell should be given to the person who found it first. In the famous case of Pierson and Post (1805) in the United States, Pearson shot a fox first while Post was chasing after it. The court finally awarded the fox to Pearson. This case demonstrated the important role of first possession in legal judgment. Psychological research showed that first possession also exists in people’s minds as a heuristic underlying ownership decisions [12–14]. For example, when subjects were shown two agents playing with toys one after the other, adults and preschoolers often assigned the toys to the first player but not the second player [12]. This has been confirmed with samples from different countries, e.g., Canada [12, 13], England [15] and Germany [16]. In addition, young children seem to have a first possessor bias because when the toys were transferred in some legal way (e.g., giving), 3-year-olds favored the original possessor but not the receiver as owner [1, 17]. A study with American 7- to 10-year-olds as subjects found that first possessor bias might persist until 7 to 8 years old [18]. Some studies found at approximately 5~9 years old, Dutch and American children apply the first possession heuristic to land [19–21] and some intellectual items (e.g., idea) [22, 23].

First possession may conflict with other cues in property disputes sometimes, which affects the stability of first possession in ownership attribution. For example, some research found that the agents’ gender and age can affect British young children’s use of the first possession heuristic [15]. Three-year-olds were more likely to assign a ball to a boy rather than to a girl and to assign a laptop to an adult rather than a child, even if the latter people were the first possessors in the context. Many scholars found people outweighed the role of labor over the first possession heuristic when they are conflicted. When told that a man modified another man’s gold into an artwork, American adults were more likely to judge the artwork as belonging to the modifier rather than the original owner of the gold [24]. Some studies also confirmed the susceptibility of young children to the conflict of labor cue [25–27]. Kanngiesser et al. [25] assigned some clay to British 3- and 4-year-old children and asked them to shape others’ clay into different clay models. The researchers found that when children were asked to judge who the processed models belong to, most of the children assigned them to themselves rather than the original owners. However, some study [28, 29] did not find such tendency. For example, Hook [28] told American 10-and 15-year-olds and adults a story, in which one boy borrowed a piece of wood from another boy and modified it into an eagle. They found that they did not agree ownership would be transferred after others’ modification. In addition, one recent study [29] used discovery stories as materials and asked Argentina 4~8-year-old children to reason ownership when pitting the first possession heuristic against the creative labor. The result showed that children supported the first possessor more than the creator.

Some research investigated non-Western children’s ownership judgments based on first possession heuristic, and found some cross-cultural differences. Kanngiesser et al. [16] compared Kikuyu children’s ownership reasoning in East Africa with German children and found that Kikuyu children did not judge ownership according to the first possession heuristic until 9 years old, similar to 5-year-old children in Germany. Kanngiesser et al. [26] showed four-year-olds from three countries (China, Japan, UK) some videos of robot-human interactions. In the videos an agent (the human or the robot) picked up a blank piece of paper after which the other agent held the paper for a few seconds. Subjects were asked to answer whose is the paper. They found whether the human or the robot held the paper first, children from three countries attributed ownership to the first holder equally. They also set a labor condition in the study. In the labor condition, the second agent drew a picture on the paper. The result showed while most British children tended to assign ownership to the laborer, Chinese and Japanese children support neither the original possessor nor the laborer as owner. In another study, Kanngiesser et al. [27] found British 3- to 4-year-old children were more likely to support the creator as the owner when they witnessed the original possessor conflicts with the laborer for the modified item, but Japanese 3- to 4-year-old children did not show such tendency.

There are still some opening questions existing in previous studies that warrant us to address. Firstly, most of studies exploring children’s first possession heuristic in the experimental settings in which one agent holds the toy first after which the other agent holds it [12–16]. There is not any background information about how the first possessor gets the object, which is different from the real ownership conflict situation related to first possession. In reality, it’s common that one person discovered one object, and another also wants it. But this situation has been ignored in most of the previous studies. One exception is Rochat et al.’s study [30]. In the study, they used two discovery stories and examined 3-year-olds’ and 5-year-olds’ ownership judgments based on the first possession heuristic in four countries (China, the United States, Brazil and Vanuatu). In the context, one of the two protagonists found a toy first under a tree and the other protagonist also wanted the toy. The researchers found that even up to five years old, children across all four countries did not judge ownership according to the first possession heuristic. The result is obviously inconsistent with most previous studies.

Secondly, when it comes to the conflict between first possession and labor, participants were often initially told who owned the object. This did not purely reflect the conflict between first possessor and laborer but reflect the conflict between initial owner versus laborer. For example, in Kanngiesser’s study [25], children were initially told they owned one lump of clay, and that the experimenter owned the other. They were required to make an animal model out of each other’s clay. The study only affirmed the original ownership of the clay, but did not provide an explicit first possession cue. In another study conducted by Kanngiesser et al. [27], children were told that the original material was theirs and they could keep it and take it home. In Hook’s study [28], subjects were explicitly told that a protagonist owned a block of wood at the beginning of the story. Therefore, there is no obvious conflict between first possession cue and labor cue.

Finally, although some research have investigated Eastern children’s ownership reasoning, the general development trend is unclear. For example, Kanngiesser et al. [26] has involved Chinese, Japanese and British preschoolers as subjects, but only included one age group (4-year-olds). Kanngiesser et al. [27] has involved Japanese and British 3-year and 4-year preschoolers as subjects, but lacked the older samples. Although Rochat et al. [30] used the discovery context and involved Chinese 3-year-olds and 5-year-olds as subjects, but we did not know whether they will use the first possession heuristic to reason ownership by six years old.

## Current study

To summarize, most previous studies have investigated preschoolers’ ownership reasoning based on first possession heuristic, but less have explored it in the discovery context. While some research examined children’s use of first possession heuristic conflicting with other cues such as labor, the design reflects the conflict between initial owner versus laborer somewhat. A clear first possession cue should be indicated. In addition, most studies that involved Eastern preschoolers focused in the younger age groups. This is not helpful for us to understand how the first possession heuristic develops. This study recruited 3-6-year-old Chinese preschoolers as subjects and adopted two discovery stories to investigate how Eastern preschoolers would respond to the ownership question when there was only the first possession cue and when there was the conflict between first possession versus labor.

To achieve this goal, we referred to Rochat et al. [30]’s study that used discovery stories as materials. We set two conditions in the stories, i.e., the single first possession cue condition and the labor condition. In the single first possession cue condition, one protagonist first discovered some objects (shell, wood) but the other protagonist also wanted it. In the labor condition, the other protagonist modified the discovered objects into some new items (shell whistle, falcon). Children were asked to answer three questions concerning ownership after each condition. As with most previous studies, we expect that children would use first possession heuristic to reason ownership at an early age (in the single first possession cue condition) but they would outweigh the labor rule over the first possession heuristic when they are conflicted (in the labor condition).

## Study 1

### Methods

The study was conducted with the approval of the Scientific Research Ethics Committee of Xi’an Jiaotong University. All subjects’ parents were asked to sign an informed consent form before children participated in the experiment.

#### Subjects

One hundred and twenty Chinese preschoolers were tested. They were thirty 3-year-olds (*M*_age_ = 3;6 [years; months], range = 3;1–3;11, 15 boys), thirty 4-year-olds (*M*_age_ = 4;7, range = 4;0–4;11, 14 boys), thirty 5-year-olds (*M*_age_ = 5;6, range = 5;0–5;11, 17 boys) and thirty 6-year-olds (*M*_age_ = 6;5, range = 6;0–6;11, 13 boys). The preschoolers were recruited from a kindergarten in Kunming. Kunming is a large city in Southwest China, with a permanent population of 6.85 million. All the subjects were of Chinese nationality and spoken Mandarin. Most of the participants came from middle to high socioeconomic backgrounds, although the detailed demographic information was not systematically collected. After the study, children received a sticker as a prize.

#### Materials and procedure

**Fig 1:**
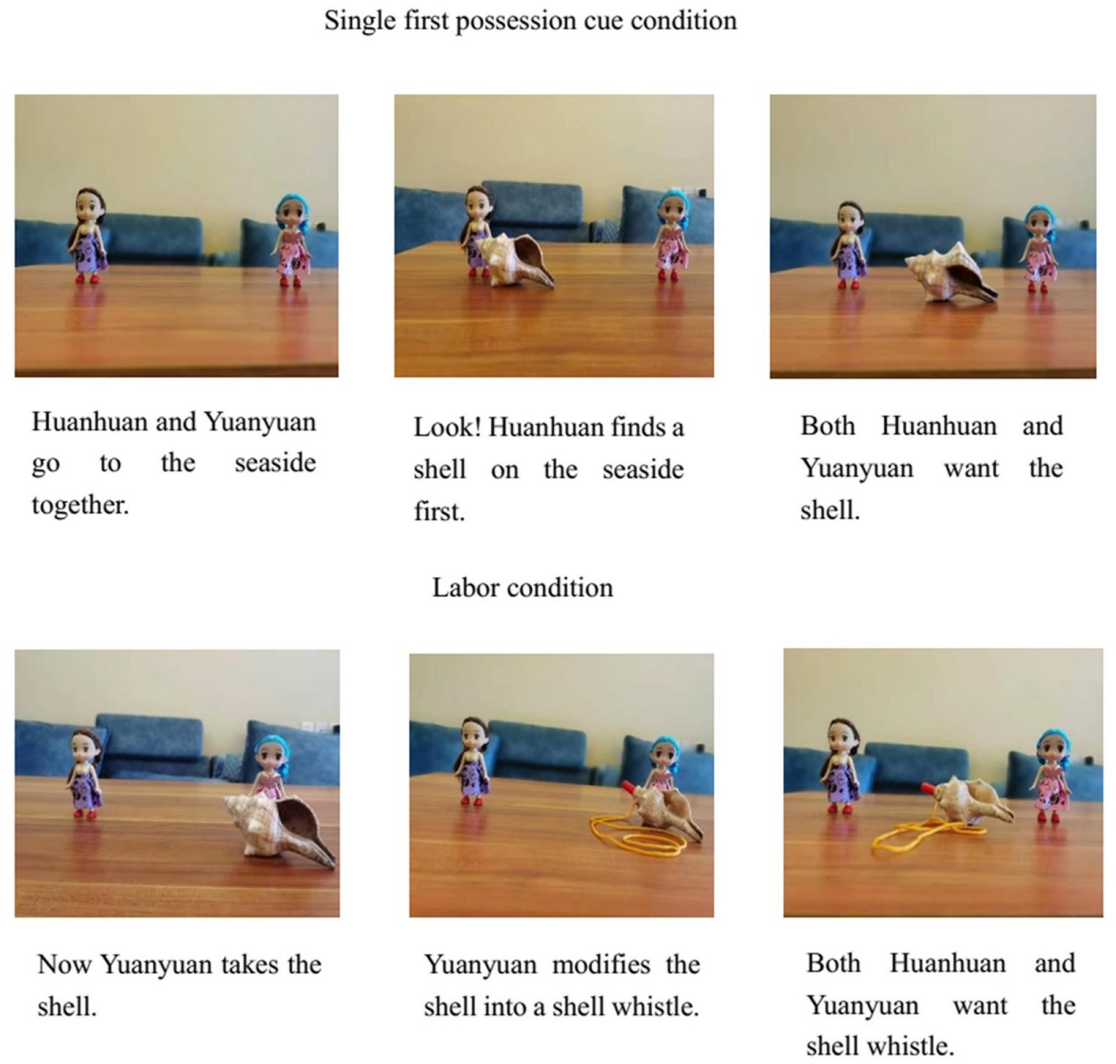
Procedures and scripts about the shell story in Study 1.

Children were individually tested in a quiet room in their kindergarten. A female postgraduate volunteering as the experimenter first played with the children for approximately one to two minutes to help them acclimate. Then, the experimenter told the children a story that was accompanied by a model demonstration. In one story (*shell story*), the experimenter put a shell and two female toy protagonists on the table. The experimenter told the subjects that the protagonists went to the seaside together, and one protagonist discovered the shell first, but both protagonists wanted it. Next, subjects were asked three ownership questions about the single first possession cue condition. *(1) Who should the shell be given to*?, *(2) Who can take the shell home*?, *and (3) Can you put the shell near her*? After those questions, the experimenter put forward a shell whistle and told subject that now the other protagonist took the shell and modified it into a shell whistle, and both protagonists wanted it. Finally, the children were asked three ownership questions about the labor condition. *(1) Who should the shell whistle be given to*?, *(2) Who can take the shell whistle home*?, *and (3) Can you put the shell whistle near her*? Due to the plot design of the story, the first possession cue was always presented before the labor cue. Therefore, questions concerning the single first possession cue condition were always asked first and questions concerning the labor cue condition were asked after that. See Fig 1 for procedures and scripts in two conditions about this story.

In another story (*wood story*), the experimenter put a piece of wood and two male toy protagonists on the table. The experimenter told subject that the protagonists went to the forest together, and one protagonist discovered the wood first, but the other protagonist also wanted it. Next, the children were asked the same three ownership questions as the shell story in the single first possession cue condition. Then, the experimenter put a falcon on the table and told subject that now the other protagonist took the wood and modified it into a falcon, and both protagonists wanted it. Finally, the subjects were asked the same three ownership questions as the shell story in the labor condition.

Subjects were forced to choose between the two protagonists in the stories. Before the ownership questions in the single first possession cue condition, the subjects were first asked a memory check question “who discovered the shell/wood first?”. Similarly, the subjects were asked another memory check question “who modified the shell/wood?” before being asked the ownership questions in the labor condition. If children failed to answer these questions, they were told the story repeatedly until they answered the question correctly. There were one 3-year-olds and one 4-year-olds needing a repetition in the shell story and two 3-year-olds needing a repetition in the wood story. To control the possible protagonist preference effect, the people who discovered the shell/wood first and people who modified the shell/wood were counterbalanced. The order of presenting the two stories was randomized. Subjects were coded 1 if they chose the discoverer and coded 0 if they chose the modifier in each ownership question.

### Results

Preliminary analysis showed that there was no significant gender effect and story effect. Therefore, the data of different genders and stories were combined for further analysis. Generalized linear models (binary logistic, Hybrid method) with age group and condition as factors entering into the model was used to examine whether children’s choices differed across different age groups and conditions. When there was interaction, pairwise comparisons tested whether there were significant age effects in the choices in each condition. Single-sample tests then examined whether children’s choices significantly above or below the expected value (50%) in each condition.

**Fig 2:**
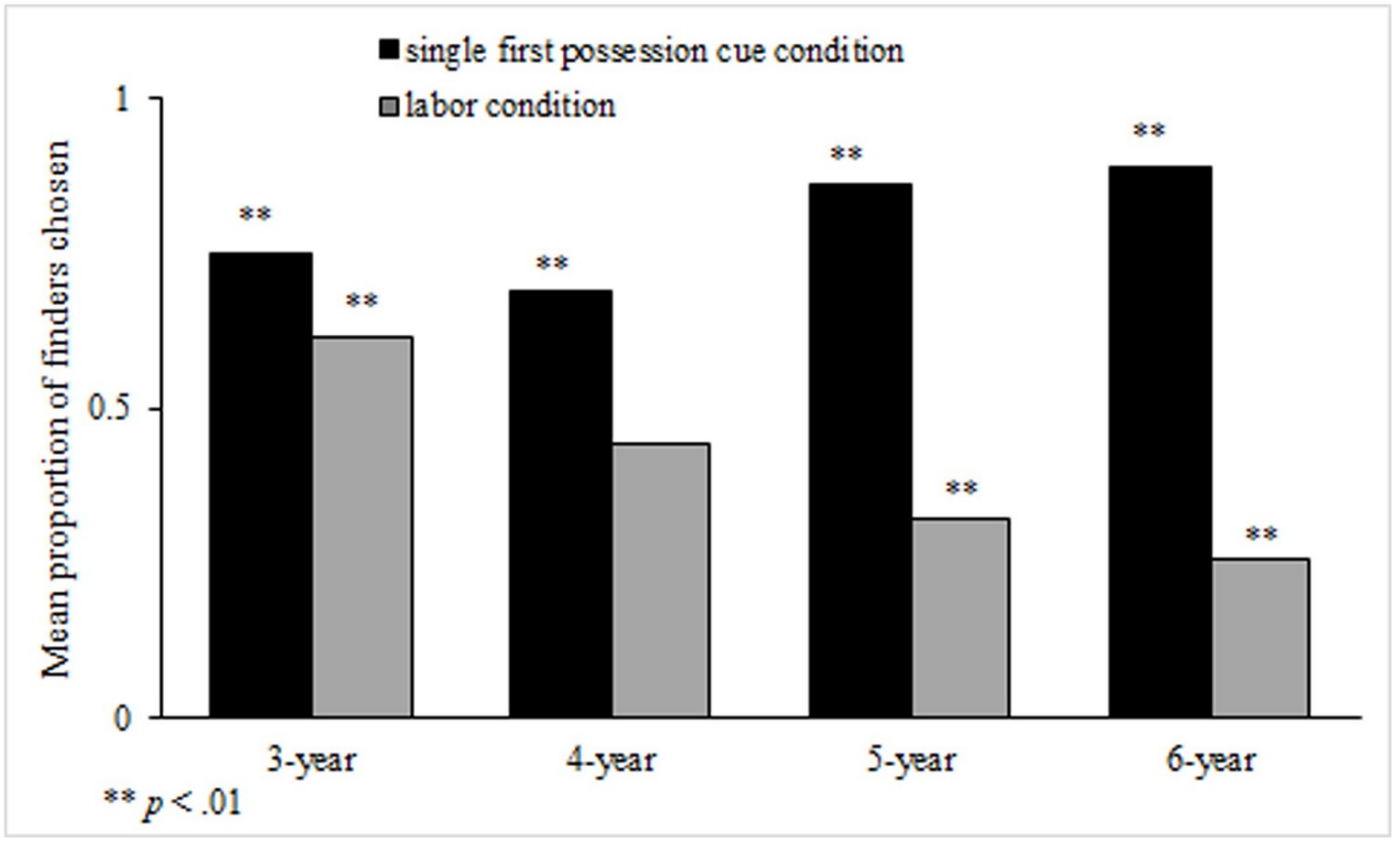
Choices of discoverers over modifiers in Study 1.

Omnibus test showed that the model effect was significant, χ^2^(7) = 320.04, *p* < .001. Children’s choices differed across age groups, Wald χ^2^(3) = 9.92, *p* < .05, and conditions, Wald χ^2^(1) = 209.12, *p* < .001, but the effects were qualified by an interaction between condition and age group, Wald χ^2^(3) = 66.03, *p* < .001. Both in the single first possession cue condition and in the conflicting cue condition, there was a significant age effect (*p*s < .001). In the single first possession cue condition, 3-year-olds and 4-year-olds chose the discoverer significantly less than 6-year-olds, and 4-year-olds chose the discoverer significantly less than 5-year-olds (*p*s < .05). However, in the labor condition, 3-year-olds chose the modifier significantly more than 4- to 6-year-olds (*p*s < .05). In addition, 4-year-olds chose the modifier significantly more than 6-year-olds (*p* = .018). In the single first possession cue condition, children in each age group chose the discoverer significantly more than expected value, *p*s < .001. In the labor condition, only 3-year-olds chose the discoverer significantly more than expected value, *p* = .011. Five-year-olds and six-year-olds chose the modifier significantly more than expected value, *p*s < .001. Four-year-olds responded at chance when choosing between the discoverer and the modifier (see Fig 2).

### Discussion

In study 1, we set two conditions, i.e., the single first possession cue condition and the labor condition, to explore Chinese preschoolers’ use of first possession heuristic to reason ownership. We found in the single first possession cue condition, children’s choices in each age group were significantly above expected value; in the labor condition, two older children’s choices were significantly below expected value, but the younger children’s choices were not. This indicates that from three years old children have the first possession heuristic in their mind, but by the age of five they began to emphasize the role of labor over first possession.

One question in study 1 is that the two conditions were not tightly matched because laborers did something to the original objects but the first possessor did not. In addition, the questions concerning first possession cue was always asked before the questions concerning labor cue. This might lead the carry-over effect that children’s responses in the labor cue condition were influenced by their answers in the single first possession cue condition. A third question in Study 1 is that we asked children only one memory check question before the ownership questions in each condition, which might imply the right answer. We would address these problems in Study 2.

## Study 2

In Study 2, we set a *first possession versus play* group and a *first possession versus labor* group. While the first possession versus labor group was the same as Study 1 except the order of two conditions was counterbalanced, the first possession versus play group involved a single first possession cue condition and a play condition. In the play condition, the other protagonist was depicted as playing with it rather than modifying it. Play was a proper control condition because it can exclude the possibility that children assign ownership just according to who did something to them. Some previous study also set the play condition as the comparison of labor condition [27]. We expect that subjects in this study would assign ownership to the first possessor in the play condition, but they would assign ownership to the laborer in the labor condition.

### Methods

The study was conducted with the approval of the Scientific Research Ethics Committee of Xi’an Jiaotong University. All subjects’ parents were asked to sign an informed consent form before children participated in the experiment.

#### Subjects

We tested 288 preschoolers. They were divided into two groups, i.e., the *first possession versus labor* group and the *first possession versus play* group. In the *first possession versus labor* group, there were 144 preschoolers. One 3-year-old preschooler and one 4-year-old preschooler were excluded from the formal analysis because they did not pass the memory control questions (see *Materials and procedure*). Therefore, the final subjects involved thirty-five 3-year-olds (*M*_age_ = 3;7 [years; months], range = 3;1–3;11, 18 boys), thirty-five 4-year-olds (*M*_age_ = 4;7, range = 4;0–4;11, 17 boys), thirty-six 5-year-olds (*M*_age_ = 5;6, range = 5;0–5;11, 17 boys) and thirty-six 6-year-olds (*M*_age_ = 6;5, range = 6;0–6;11, 18 boys). In the *first possession versus play* group, there were 144 preschoolers. Two 3-year-old preschooler and one 4-year-old preschooler were excluded from the formal analysis because they did not pass the memory control questions. Therefore, the final subjects involved thirty-four 3-year-olds (*M*_age_ = 3;6 [years; months], range = 3;0–3;11, 17 boys), thirty-five 4-year-olds (*M*_age_ = 4;5, range = 4;0–4;11, 18 boys), thirty-six 5-year-olds (*M*_age_ = 5;5, range = 5;0–5;11, 18 boys) and thirty-six 6-year-olds (*M*_age_ = 6;6, range = 6;0–6;11, 18 boys). All preschoolers were recruited from the same kindergarten as Study 1.

#### Materials and procedure

The same models and toys as Study 1 were used as materials in this study. Children were tested in the same room and by the same female postgraduate who was blind to the hypothesis. Due to the influence of COVID-19, Study 2 was conducted nearly eight months later after the Study 1 when China’s COVID-19 has been greatly alleviated.

#### *First possession versus labor* group

We told subjects the same stories as Study 1 and demonstrated the scene with the same models, but the questioning order of two conditions was counterbalanced. In the cases where questions about first possession cue condition were presented before the labor cue condition, subjects were asked questions in the same way as Study 1. That is, they were asked questions below after hearing that the first possessor discovered the shell: *(1) Who should the shell be given to*?, *(2) Who can take the shell home*?, *and (3) Can you put the shell near her*?. Then they were asked *(4) Who should the shell whistle be given to*?, *(5) Who can take the shell whistle home*?, and *(6) Can you put the shell whistle near her*? after hearing the whole story. In the case where questions about labor condition presented before the single first possession cue condition, subjects were asked questions after hearing the whole story. First they were asked *(1) Who should the shell whistle be given to*?, *(2) Who can take the shell whistle home*?, and *(3) Can you put the shell whistle near her*? Then they were asked *(4) Who should the shell be given to before it was modified*?, *(5) Who can take the shell home before it was modified*?, *and (6) Can you put the shell near her*? Half of the subjects were asked the questions about the single first possession cue condition first and the other half were asked the questions about the labor cue condition first. After answering all ownership questions, subjects were asked two memory check questions, (1) who discovered the shell first?, (2) who has modified the shell?. Children who failed to answer these two questions were not included into the final analysis.

#### *First possession versus play* group

**Fig 3:**
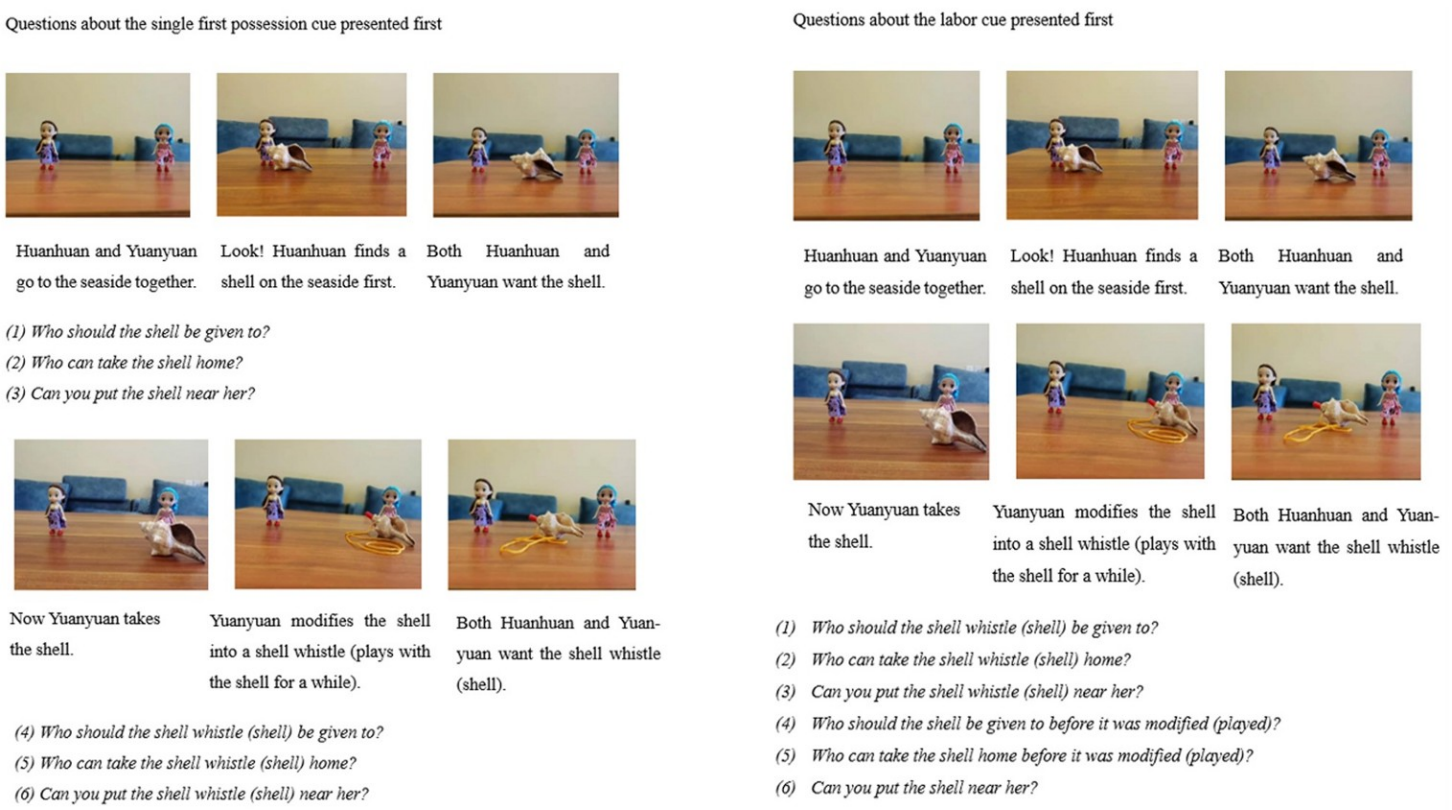
Procedures and scripts about the shell story in the *First possession versus labor (play)* group in Study 2.

In this group, the other protagonists were depicted as just playing with the shell for a while instead of modifying it. As with the *first possession versus labor* group, subjects were asked three questions about the single first possession cue condition and three questions about the play condition, with half answering questions about the single first possession cue condition first and the other half answering questions about the play cue condition first. They were asked two memory control questions to check the understanding of the story. Fig 3 shows procedures and scripts about the shell story in two groups. The same procedure applies to the wood story, but the shell in the script was replaced with wood and the shell whistle with falcon. As with Study 1, protagonists who discovered the shell/wood first and protagonists who modified the shell/wood were counterbalanced. The order of the two stories presented was randomized.

Subjects were coded 1 if they chose the discoverer and coded 0 if they chose the modifier (in the *First possession versus labor* group) or the player (in the *First possession versus play* group) for each ownership question.

### Results

No significant gender effect and story effect were revealed in the preliminary analysis, data from different genders and stories were combined in the subsequent analysis. There was also no significant questioning order effect in the study. Generalized linear models (binary logistic, Hybrid method) was conducted with group, age and condition as factors entering into the model. Omnibus test showed that the model effect was significant, χ^2^(15) = 525.73, *p* < .001. Children’s choices differed across groups, Wald χ^2^(1) = 90.40, *p* < .001, and ages, Wald χ^2^(3) = 9.11, *p* = .028, and conditions, Wald χ^2^(1) = 123.02, *p* < .001.

The interaction between group and age was significant, Wald χ^2^(3) = 17.53, *p* = .001. Pairwise comparisons showed there were both significant age effects in two groups, *p*s < .01. Four- to six-year-olds were more likely to choose the discoverer in the *First possession versus play* group than in the *First possession versus labor* group, *p*s < .01, but there was no significant group effect in the 3-year-olds. The interaction between group and condition was significant, Wald χ^2^(1) = 97.71, *p* < .001. In the *First possession versus labor* group, children were more likely to choose the discoverer in the single first possession cue condition than in the labor cue condition, *p* < .01. However, in the *First possession versus play* group, children’s choices did not differ between the two conditions. For the single first possession cue condition, no significant group effect was revealed; but for the labor cue condition, children’s choices for the discoverer were significantly more in the *First possession versus play* group than in the *First possession versus labor* group, *p* < .01. The interaction between age and condition was significant, Wald χ^2^(3) = 37.92, *p* < .001. There were both significant age effects in the two conditions, *p*s < .01. Four- to six-year-olds were more likely to choose the discoverer in the single first possession cue condition than in the labor cue condition, *p*s < .01, but there was no such difference in the 3-year-olds. In addition, the three-way-interaction was also revealed, Wald χ^2^(3) = 32.07, *p* < .001. Therefore, we split the group and analyzed the effect of interaction between age and condition in each group separately. We found that the effect of interaction between age and condition was significant in the *First possession versus labor* group, Wald χ^2^(3) = 72.65, *p* < .001, and the results of pairwise comparisons were similar as above analysis when group was not split, but the difference was not significant in the *First possession versus play* group, Wald χ^2^(3) = 0.30, *p* = .96.

**Fig 4:**
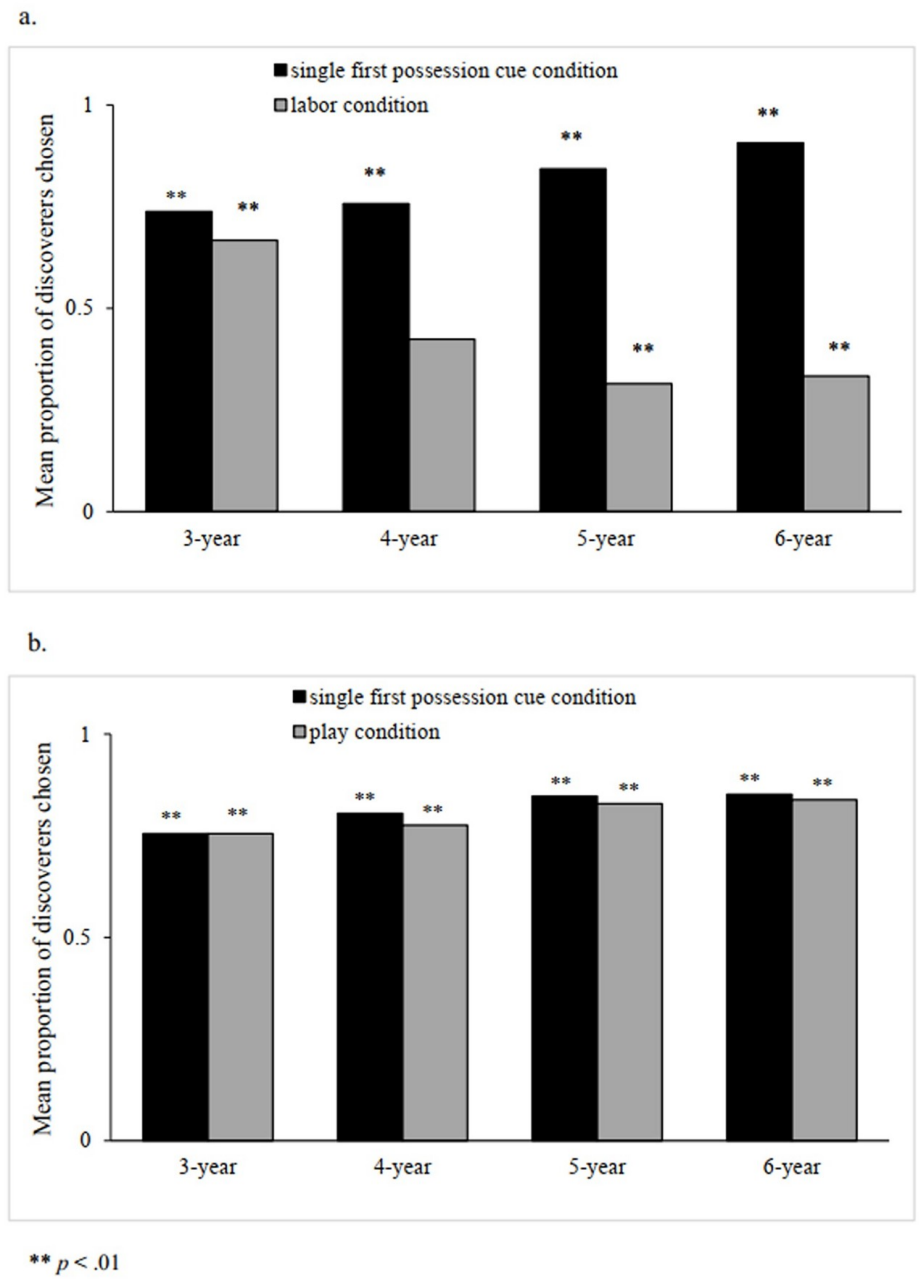
Choices of discoverers over modifiers in Study 2. a. *First possession versus labor* group, b. *First possession versus play* group).

Single-sample tests showed that for the *First possession versus labor* group, children in each age group chose the discoverer significantly more than the expected value in the single first possession cue condition, *p*s < .001; however, in the labor condition, only 3-year-olds chose the discoverer significantly more than expected value, *p* = .011, whereas five-year-olds and six-year-olds chose the modifier significantly more than expected value, *p*s <. 01. Four-year-olds responded at chance when choosing between the discoverer and the modifier. For the *First possession versus play* group, in both the single first possession cue condition and the play condition, children in each age group chose the discoverer significantly more than expected value, *p*s < .001 (see Fig 4).

### Discussion

Study 2 set a *first possession versus play* group and a *first possession versus labor* group to investigate children’s ownership judgments further. The results showed a significant interaction between age and condition in the *First possession versus labor* group. This is consistent with the results of Study 1. But the interaction between age and condition was nonsignificant in the *First possession versus play* group. Moreover, for the *First possession versus labor* group, children in each age group chose the discoverer significantly more than the expected value in the single first possession cue condition, but five-year-olds and six-year-olds chose the modifier significantly more than expected value in the labor condition. However, for the *First possession versus play* group, children of all age groups chose the discoverer significantly more than expected value in the single first possession cue condition as well as in the play cue condition. These results indicates that older preschoolers would give up their support for the discoverer when the first possession cue conflicts with the labor cue, but they would still support the discoverer when the first possession cue conflicts with the play cue. The result ruled out the possibility that older preschoolers’ support for the discoverer was due to the fact that laborers do something to the original objects but the first possessors do not.

## General discussion

This study designed two discovery stories to investigate young Chinese children’s ownership reasoning with the first possession heuristic and its stability when it is conflicted with labor cue. With two studies, we found children’s ownership judgments rarely changed from 3 to 6 years old in the single first possession cue condition. Even 3-year-old children judged ownership based on the first possession heuristic, as their choices for the discoverer significantly above expected value, which was similar to the results observed in the older children. However, in the labor condition where the labor cue conflicted with the first possession cue, choices for the discoverer decreased sharply from 3 to 6 years old. While 3-year-old children still insisted on using the first possession heuristic, 4-year-old children became swayed because their choices were not above or below expected value significantly, and 5- to 6-year-olds turned to support the modifiers in the stories. In contrast, in the play condition in study 2, there were no significant changes in children’s choices across four age groups. This confirms that labor is an important consideration in young children’s ownership reasoning which can affect the stability of first possession heuristic. The results demonstrate that in the discovery context Chinese preschoolers make ownership judgments according to the first possession heuristic at an earlier age, and with development, older children are not as insistent on the first possession heuristic as the youngest preschoolers when there are some conflicting cues such as labor.

An important difference between this study and previous studies is that we used discovery contexts to investigate children’s adoption of the first possession heuristic. Many previous studies [12–15] did not provide the background information of how the first possessors obtain the objects. One exception is Rochat et al.’s study [31], which used the discovery stories to investigate 3- and 5-year-olds’ ownership reasoning with different cues. They found that even up to 5 years old, children did not use the first possession heuristic to reason about ownership. The current study also used discovery stories, but the results are inconsistent with Rochat et al.’s study. We found that from 3 years old, children could judge ownership based on the first possession heuristic. It should be noted that we chose two natural items (shell, wood) as the objects discovered in the stories, but in Rochat et al.’s study, they used artificial items (i.e., toys) as the objects discovered. Some studies have shown that preschoolers in Canada often think intuitively that an artificial object is possessed by someone but a natural object was not possessed by someone [31]. Even being supplied with the location information, they would likely reason an artificial object being owned whether it was located indoors or outdoors [32]. Subjects might regard the discovered toy was just lost by someone else thus would not like to assign it to any one of the protagonists. The adoption of natural objects avoids this possibility such that the first possession heuristic was detected earlier. Future studies can examine this possibility by distinguishing the natural objects from the artificial objects in the scenarios.

In this study, we introduced an explicit first possession cue and pit it against the labor cue. Such design was different from previous studies that preassign an owner of the original object which was then modified by the laborer. We found facing with the first possession versus labor conflict, younger preschoolers (3-year-olds and 4-year-olds) did not like to forfeit the first possession heuristic. This finding was consistent with Kanngiesser et al.’s study [26, 27] which found Japanese 3- to 4-year-olds and Chinese 4-year-olds did not show obvious preference for the laborer over the first possessor. Both studies found British young preschoolers were more likely to choose the laborer rather than the first possessor. This study further evidenced the cross-cultural difference in the stability of children’s use of the first possession heuristic. This finding might be due to the specific parenting style in Asian countries. Asian parents often show more strict discipline and overprotection to their children [33]. This parenting style may limit children’s self-exploratory behavior and the amount of time children spent with new objects [34], which may further limit the awareness of labor’s role in ownership.

The study extended the age period investigated and found that by 5 years old, Chinese children gave up first possession bias and weighed the role of labor in the labor condition. Two mechanisms may contribute to this shift. First, with the development of perceptual motor ability, children are increasingly able to exert influence on objects through labor, which leads them to pay more attention to the role of labor in ownership. Second, through peer interaction, children gradually become aware that a harmonious relationship is more important than the insistence of the first possession rule. This awareness may help them learn to abandon ownership in some contexts and enhance friendship quality.

## Limitation and future studies

This study assumed that the modifiers directly took the items but did not declare the legitimacy of the taking behavior (e.g., being permitted, borrowed or stolen). It’s commonly acknowledged that the legitimacy of an obtaining object will affect ownership assignments between the original owner and the obtainer. A recent study [35] examined how Chinese adults assign ownership when someone’s objects are acquired and modified by another person in different ways (kept, borrowed, found). It was found that adults believed that the modified objects still belong to the original owner when they were just kept by the modifier, but they belong to the modifier when they were lost by the original owner and found by the modifier. This suggests that adults’ ownership judgments based on first possession heuristic are affected not only by the labor cue, but also by the transfer way of objects. To our knowledge, no research has explored how children address such issues. Several studies have shown that preschoolers could distinguish the legal transfer (e.g., give) from the illegal transfer (e.g., steal) of ownership from the age of five [1, 17]. Future research may design appropriate method to detect how they weigh the role of first possession heuristic, labor and transfer way of objects in ownership assignments.

## Supporting information

S1 File (TXT)

S1 Data (XLSX)

S2 Data (XLSX)
